# Maternal Different Degrees of Iodine Deficiency during Pregnant and Lactation Impair the Development of Cerebellar Pinceau in Offspring

**DOI:** 10.3389/fnins.2017.00298

**Published:** 2017-05-29

**Authors:** Jing Dong, Heling Song, Yuan Wang, Min Li, Ye Yu, Yi Wang, Jie Chen

**Affiliations:** Department of Occupational and Environmental Health, School of Public Health, China Medical UniversityShenyang, China

**Keywords:** iodine deficiency, cerebellum, purkinje cells, axon initial segment, pinceau

## Abstract

**Aims:** Iodine is critical for synthesis of thyroid hormones (TH). And iodine deficiency (ID) is one of the most significant reasons of intellectual disability and motor memory impairment, although the potential mechanisms are still under investigation. Presently, mild ID and marginal ID are largely ignored problems for women of child bearing age. Mild ID is a subtle form of TH deficiency, which shows low levels of free thyroxine (FT_4_) and relatively normal free triiodothyronine (FT_3_) or thyroid stimulation hormone (TSH). And marginal ID is a milder form of ID with decreased total T_4_ (TT_4_) but relatively normal FT_3_, FT_4_, and TSH. Therefore, we investigated the effects of maternal different degrees of ID on the development of pinceau in cerebellar purkinje cells (PCs) and studied the expression of pinceau related protein, which is crucial for the development and maturation of pinceau.

**Methods and Results:** Three developmental iodine deficient rat models were created by feeding dam rats with an iodine-deficient diet and deionized water supplemented with potassiumiodide. Our study showed that different degrees of ID inhibited cerebellar pinceau synapse development and maturation on postnatal day (PN) 14 and PN21. What's more, mild and severe ID reduced the expression of AnkG, β4-spectrin, neurofascin186 and NrCAM on PN7, PN14, and PN21. However, marginal ID rarely altered expression of these proteins in the offspring.

**Conclusion:** These results suggested that maternal mild and severe ID impaired the development and maturation of cerebellar pinceau, which may be attributed to the decrease of AnkG, β4-spectrin, neurofascin 186, and NrCAM. And the alteration of development and maturation in cerebellar pinceau in the offspring were also observed following maternal marginal ID, which is slighter than that of mild ID.

## Introduction

Iodine is widely presents in nature. As an essential micronutrient for human health, iodine is critical for the synthesis of thyroid hormones (TH). Biochemically, iodine is drawn into the thyroid and combined the tyrosyl residues of thyroprotein to ultimately form triiodothyronine (T_3_) and thyroxine (T_4_). It is well-known that iodine deficiency (ID) can lead to irreversible neurodevelopment impairments, cognitive function defection, psychomotor, and motor dysfunction (Berbel et al., 2007; Tang et al., 2007; Walker et al., 2007; Williams, 2008), which is the widespread health problem. Highly effective strategy of universal salt iodization has been taken in China to control severe ID. However, as to special populations—pregnant and lactating women, it is likely to be a shortage of iodine supplementation in their body, due to the increased physiological demands for nutritional iodine (Gärtner, 2009). The World Health Organization (WHO) recommends an iodine intake of 250 μg/day and suggests median urinary iodine concentrations (UIC) of 150–249 μg/l to prevent iodine deficiency disorders in pregnancy (World Health Organization/UNICEF/ICCIDD, 2007). Even in some developed countries and regions, mild ID, and marginal ID are largely ignored problems for women of child bearing age (Morreale de Escobar et al., 2000; Hollowell and Haddow, 2007; Perrine et al., 2010; Pearce et al., 2013). Monitoring data have indicated that a large percentage of women during pregnancy and lactation have UIC of 100–150 μg/l in the developing country, which is slightly lower than the WHO criteria for sufficient iodine supply (World Health Organization/UNICEF/ICCIDD, 2007; Ferreir et al., 2014; Méndez-Villa et al., 2014). Mild ID is identified as one of the most frequent causes of maternal and neonatal hypothyroxinemia (Morreale de Escobar et al., 2000; Vermiglio et al., 2004; Stagnaro-Green et al., 2011; Henrichs et al., 2013). Hypothyroxinemia shows low levels of free thyroxine (FT_4_) and relatively normal free triiodothyronine (FT_3_) or thyroid stimulation hormone (TSH), which is a subtle form of TH deficiency. While, marginal ID is milder than mild ID, which shows decreased total T_4_ (TT_4_) but relatively normal circulating levels of FT_3_, FT_4_, and TSH (Versloot et al., 1998). This is alarming given that it is necessary to pay more attention to the effect of the mild thyroid hormone change on the neurodevelopment of the offspring.

The cerebellum was reported to play a vital role in motor activity and motor coordination, the function of which depends on sufficient TH (Brooks, 1984). Our previous research findings suggested that hypothyroxinaemia caused by mild ID affected dendritic development of cerebellar purkinje cells (PCs), the proliferation of cerebellar granule neuron precursors (CGNPs), and differentiation of cerebellar granular cells (Wang et al., 2014a,b; Dong et al., 2016; Min et al., 2016a). As the most important neuron in the cerebellum, PCs summate all of stimulations to the cerebellum and are the only efferent nerves of the cerebellum (Palay and Chan-Palay, 1974). Therefore, the activity of PCs should be strictly controlled. And this regulation is partly implemented by the basket cell (Sotelo, 2008; Buttermore et al., 2013), which is an inhibitory interneuron in the cerebellum. Basket cell axons branch many paraxons to the purkinje soma/axon initial segment (AIS) area and form a specialized structure, which is regarded as pinceau (Buttermore et al., 2013). The pinceau is critical for normal cerebellar development and function. It has been demonstrated that impairment of pinceau functional organization resulted in ataxia, which is a consequence of PCs dysfunction (Xie et al., 2010). Although the normal function of purkinje neuron depends on the pinceau, the AIS on PCs is also crucial to the development and functional organization of the pinceau (Ango et al., 2004; Huang, 2006; Zonta et al., 2011; Buttermore et al., 2012).

The AIS is a specialized short region in the axon. And it locates between the axon hillock and the myelin sheath in myelinated axons, which has two main functions: to initiate and modulate action potentials in mammalian neurons and to maintain neuronal polarity (Zonta et al., 2011; Yoshimura and Rasband, 2014). Functions as both a physiological and physical bridge between the somatodendritic and axonal domains, the AIS is enriched in voltage-gated ion channels, cell adhesion molecules (CAM), the cytoskeletal proteins β4-spectrin and Ankyrin G (AnkG; Szu-Yu Ho and Rasband, 2011; Yoshimura and Rasband, 2014). Among the known AIS proteins, cytoskeletal adaptor protein AnkG is the master organizer for AIS assembly (Zhou et al., 1998; Jenkins and Bennett, 2001). *In vivo* and *in vitro* experiments suggested that AnkG is required for all subsequent proteins enrichment during development and throughout life (Jenkins and Bennett, 2001; Hedstrom et al., 2008; Sobotzik et al., 2009). Lack of AnkG, the formation of AIS is disturbed and the axonal polarity is disrupted, which leads to abnormal organization of the pinceau (Sobotzik et al., 2009; Buttermore et al., 2012). Furthermore, AnkG is accompanied at the AIS by β4-spectrin, which is a specific scaffolding protein. Like other spectrins, β4-spectrin binds to the AnkG and the actin cytoskeleton (Grubb and Burrone, 2010).

Extracellular signaling molecules neurofascin 186 (NF186) and neuron related cell adhesion molecule (NrCAM) are members of the L1 family of CAMs (Ogawa and Rasband, 2008). They arise relatively late and are rich in the AIS, and depend on AnKG for locating to AIS (Jenkins and Bennett, 2001; Boiko et al., 2007; Hedstrom et al., 2007). NF186 is necessary to the functional organization and long-term maintenance of the AIS (Hedstrom et al., 2008; Zonta et al., 2011). And the purkinje neurons that loss NF186 could lead to slow disorganization of the purkinje AIS and pinceau morphology (Buttermore et al., 2012). In addition, accompanied with the distribution of NF186, GABAergic inputs to proximal axons of cerebellar PCs precisely. On the other hand, NF186 is also dependent upon the localization of AnkG (Ango et al., 2004). Taken together, the proteins AnkG, β4-spectrin, NF186, and NrCAM are participate in the guidance of basket cells along the axons of PCs and the formation of the pinceau.

Previous studies have demonstrated that ID in different degrees can affect Purkinje cells dendritic growth of the offspring (Wang et al., 2014a). Presently, there is little evidence to suggest that Purkinje AIS of the offspring is impacted by maternal marginal and mild ID. It is speculated that the impaired PCs induced by ID might be involved in the altered instruction of AIS. Consequently, the aim of this study was to illuminate the effects of maternal ID on the vital structure “pinceau” in pups cerebellum and the underlying mechanisms involved.

## Materials and methods

### Animals

Wistar rats (130–150 g) were obtained from the Center for Experimental Animals at China Medical University (Shenyang, China) with the National Animal Use License number SCXK-LN2003-0009. And all experiments and surgical procedures were approved by the Animal Care and Use Committee at China Medical University, which complies with the National Institutes of Health Guide for the Care and Use of Laboratory Animals. Rats were housed at a temperature of 24 ± 1°C with 12 h light/12 h dark cycles. Food and water were provided *ad libitum*. After acclimatized for 1 week, Female rats was administered with an ID diet (iodine content 60 ± 1.5 ng/g, measured by As^3+^-Ce^4+^catalytic spectrophotometry) and deionized water added with potassium iodide (KI) in different concentrations. And the final concentrations of iodine in the water were 0, 50, 117, and 183 μg/l in severe ID, mild ID, marginal ID, and control groups, respectively. Based on the daily intake of the 30 ml water and 25 g diet, it is estimated that the overall iodine intake of the female rats was controlled in 1.5, 3.0, 5.0, and 7.0 ug/day in the four groups. After 3 months for specific diet fed, the female rats and normal male rats were then mated (♀/♂ = 2:1). And the day was marked as gestational day (GD) 0 when the vaginal plug was observed. All the pregnant rats of four groups were fed according to the aforementioned methods till to postnatal day (PN) 21. Each group consisted of about 12 pregnant rats. And each litter was culled to about eight pups on PN4 (same number of males and females in each group, if possible). These marginal, mild, and severe ID animal models were successfully obtained in our previous researches (Dong et al., 2016; Min et al., 2016b, 2017).

### Iodine-deficient diet

The iodine-deficient diet is produced according to the AIN-93G purified rodent diet guidelines (Reeves et al., 1993). And its bases, including corn (30%), rice (30%), and soybean (40%), were obtained from an ID epidemic area and did not contain any component of animal origin. In addition, each kilogram of the iodine-deficient diet was fortified with mineral mixtures (35 g, KI excluded), vitamin mixture (10 g), L-lysine (13 g), L-threonine (6.7 g), L-methionine (4.6 g), L-tryptophan (2.1 g), L-choline (1 g), and corn oil (10 ml), which is to avoid the possible poor growth in the pups because of mothers fed with this nutritionally inadequate diet.

### Tissue collection

On PN14 and PN21, pups were conducted intracardiac perfusion with pre-cooling and containing 0.02% heparin saline (50–100 ml), and followed by 4% paraformaldehyde (about 200–400 ml) in 0.1 M potassium phosphate buffer (pH = 7.4). The cerebella were then quickly removed from the skull and fixed overnight in the same fixative. The fixed cerebella were embedded in paraffin and sectioned into 6-μm-thick sagittal sections. Sectioning was carried out in a serial manner. And every fifth/sixth slice was collected from each rat and mounted on gelatin-coated microscope slides. Three sections of each rat cerebellum were selected randomly for staining.

### Immunofluorescence

Immunofluorescence was performed as described by Dong et al. (2016). After deparaffinization and washing, the preincubated sections were incubated with the mouse monoclonal antibody anti-Calbindin-D-28 K (Sigma-Aldrich, St. Louis, MO, USA; ratio of 1:3000) and the rabbit polyclonal antibody anti-GAD65 (Santa Cruz Biotechnology, Inc., USA; ratio of 1:50), or the mouse monoclonal antibody anti-Calbindin-D-28 K and the rabbit polyclonal antibody anti-Neurofascin (Santa Cruz Biotechnology, Inc., USA; ratio of 1:25) for double immunofluorescence labeling. Then, tissue sections were washed in PBS after overnight at 4°C, and incubated for 2 h using secondary antibody conjugated to the fluorescent markers FITC and Rhodamine (Zhongshan Biotechnology, Beijing, China; ratio of 1:100). Finally, tissue sections were mounted with glycerin gelatin for histological examination. The slices labeled by FITC and Rhodamine were observed by a fluorescence microscope (BX61+DP-71; Olympus/IPP, Japan/USA). And these images were obtained from cerebellar lobule 4–5 and then merged at a magnification of × 400 (ocular × 10 and objective × 40), respectively. The mean intensities of GAD65 and NF186 in the cerebellum were obtained by using image analysis program (MetaMorph, UIC, US). Three different fields per section were selected, and three sections per animal were measured to get a mean value.

### Western blotting

Pups were deeply anesthetized and euthanized by ether on PN7, PN14, and PN21. The cerebella of pup were rapidly removed from the skulls, and then homogenized in buffered isotonic cocktail (250 μl) that contained protease and phosphatase inhibitors. Subsequently, samples were sonicated and centrifuged at 13,000 × g in 4°C for 10 min. After re-centrifugation, the finally obtained supernatants were stored at −70°C until required for analysis. Protein concentrations were estimated by Pierce BCA Protein Assay Kit (Thermo Scientific, USA). Tissue lysates of each rat were diluted to a protein concentration of 3 μg/μl. After boiled for 5 min, 10 μl aliquots sample was loaded onto 10% SDS-acrylamide gels. Then, proteins were separated using a constant voltage of 100 V and were transferred onto PVDF membranes. After blocking non-specific sites, membranes were washed and then incubated with rabbit polyclonal antibody anti-Ankyrin G (Santa Cruz Biotechnology, Inc., USA; ratio of 1:800), rabbit polyclonal antibody anti-spectrin β4 (Santa Cruz Biotechnology, Inc., USA; ratio of 1:800), rabbit polyclonal antibody anti-NrCAM (Abcam, MA, USA; ratio of 1:1000), rabbit polyclonal antibody anti-Neurofascin (Abcam, MA, USA; ratio of 1:1000), and rabbit polyclonal antibody anti-β-Tubulin (Cell Signaling Technology, MA, USA; ratio of 1:2000) at room temperature for 2 h. Then, these membranes were incubated for 1 h with goat anti-rabbit horseradish peroxidase-conjugated secondary antibody (Proteintech Group, Inc., USA; ratio of 1:2000). Using the Easy Enhanced Chemiluminescence Western Blot Kit (TransGen Biotech, Beijing, China), the blots were developed. Subsequently, protein bands were detected with an image analysis program (Gel Image Systemver. 4.00) to quantify the optical density, and data corrected for background chemiluminescence were recorded and analyzed. And β-Tubulin bands were as a loading control for each blot.

### Statistics

All data analyses were done using the SPSS software (version 16.0, SPSS Inc., Chicago, IL, USA), and all experiments were conducted in at least triplicate. The data were presented as means ± standard deviations (*SD*). And a one-way analysis of variance followed by the Student-Newman–Keuls test was applied to compare the differences in the three treated groups and control group. *P* < 0.05 was considered statistically significant.

## Results

### Different degrees of ID inhibited the development of pinceau in cerebellar PCs

**Figure 1:**
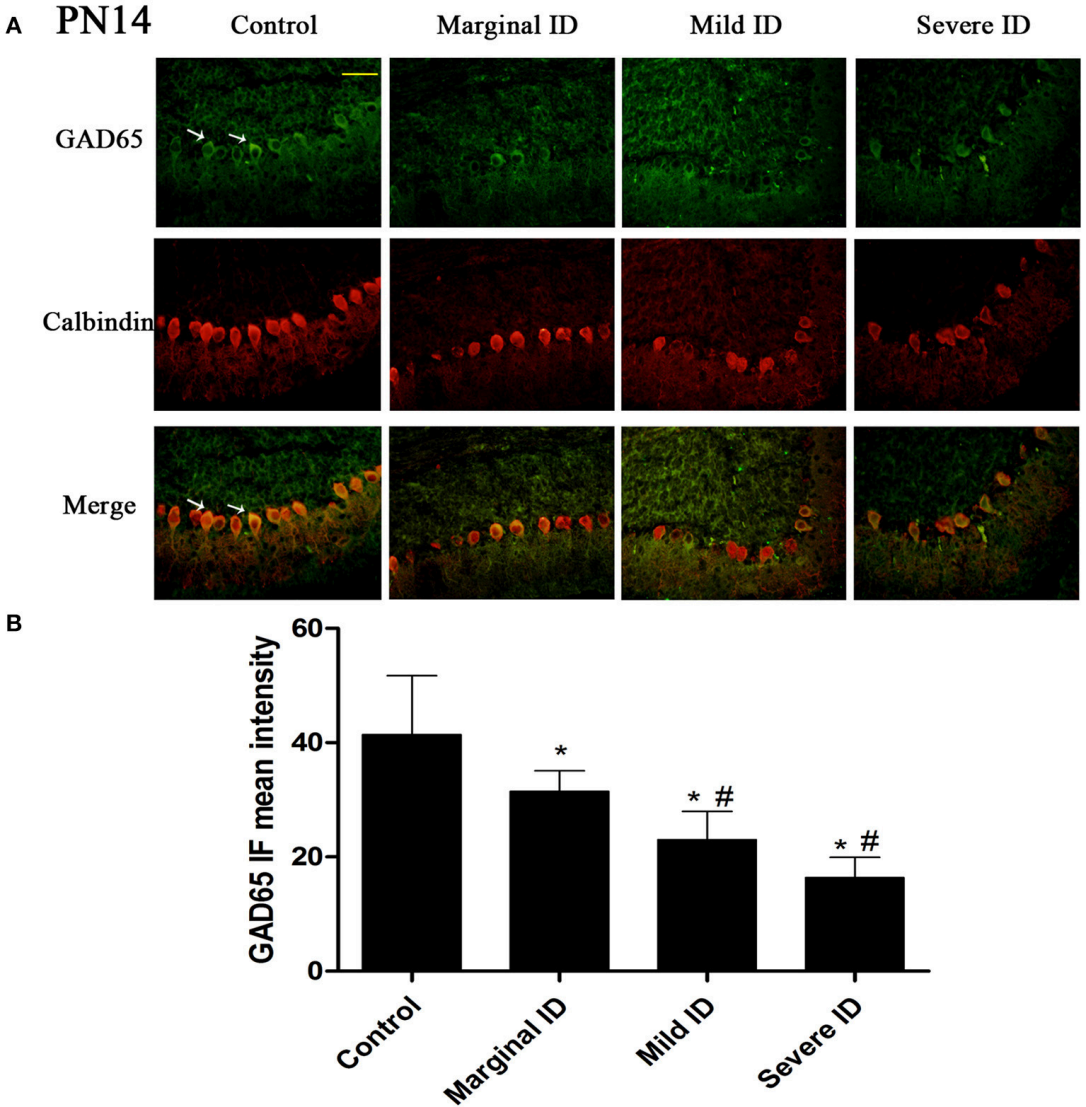
**Different degrees of ID reduced the expressions of GAD65 in cerebellar PCs on PN14**. Representative photomicrographs show fluorescent staining of anti-GAD65 (green) and anti-Calbindin-D-28 K (red) in the PCs on PN14 **(A)** (*n* = 5). The merged images show overlapping localization of these two proteins. GAD65^+^ and Calbindin^+^ (arrow) show the pinceau. Scale bar = 100 μm. The bar graphs **(B)** show the results of the mean intensity of GAD65 immunofluorescence in the four different groups on PN14. Each bar represents the mean ± *SD* for the groups. ^*^ indicates a significant difference from the control group, *p* < 0.05; # indicates a significant difference from the marginal ID group, *p* < 0.05.

**Figure 2:**
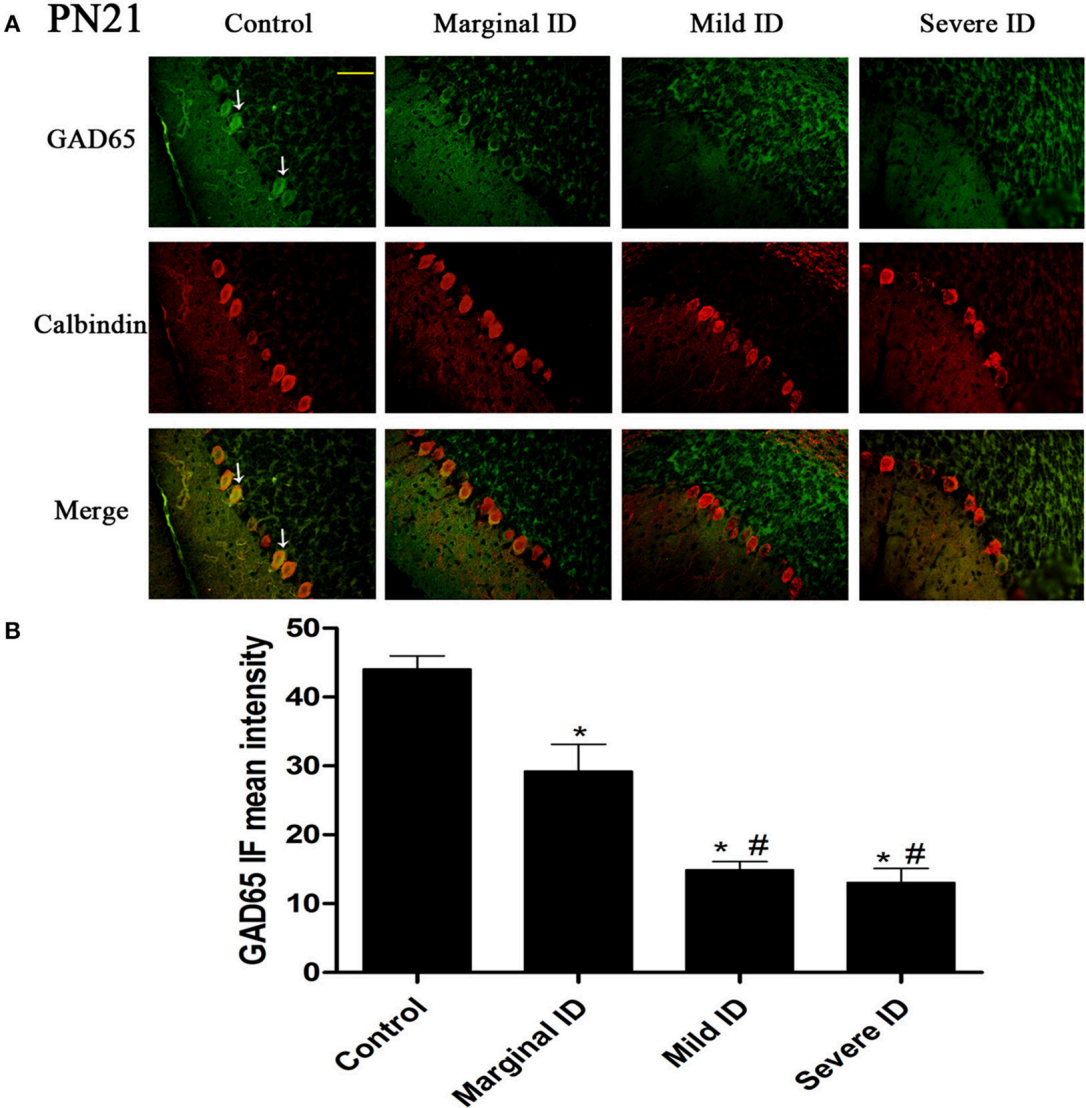
**Different degrees of ID reduced the expressions of GAD65 in cerebellar PCs on PN21**. Representative photomicrographs show fluorescent staining of anti-GAD65 (green) and anti-Calbindin-D-28 K (red) in the PCs on PN21 **(A)** (*n* = 5). The merged images show overlapping localization of these two proteins. GAD65^+^ and Calbindin^+^ (arrow) show the pinceau. Scale bar = 100 μm. The bar graphs **(B)** show the results of the mean intensity of GAD65 immunofluorescence in the four different groups on PN21. Each bar represents the mean ± *SD* for the groups. ^*^ indicates a significant difference from the control group, *p* < 0.05; # indicates a significant difference from the marginal ID group, *p* < 0.05.

**Figure 3:**
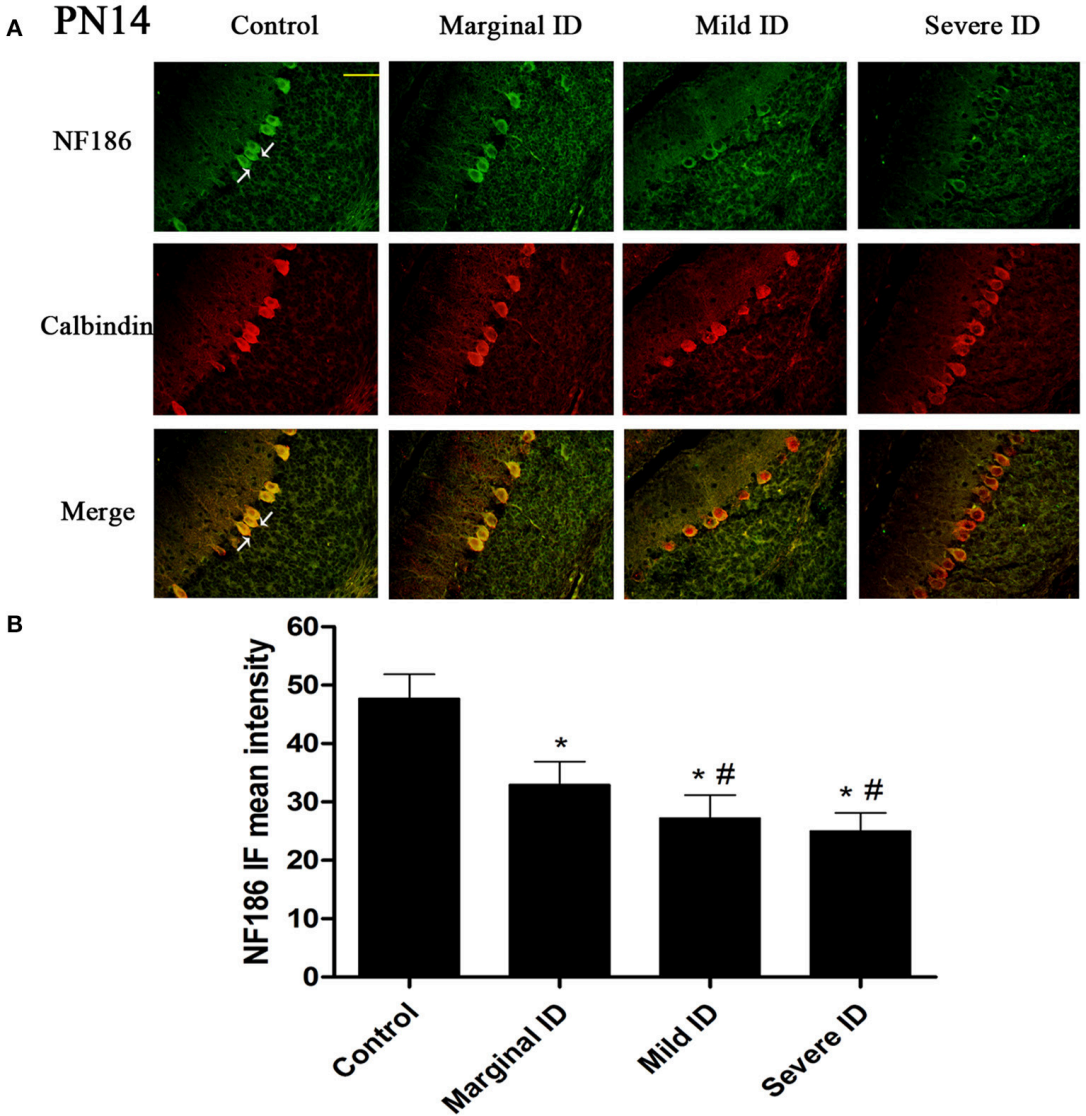
**Different degrees of ID decreased the levels of NF186 in cerebellar PCs on PN14**. Representative photomicrographs show fluorescent staining of anti-NF186 (green) and anti-Calbindin-D-28 K (red) in the PCs on PN14 **(A)** (*n* = 5). The merged images show overlapping localization of these two proteins. NF186^+^ and Calbindin^+^ (arrow) show the pinceau. Scale bar = 100 μm. The bar graphs **(B)** show the results of the mean intensity of NF186 immunofluorescence in the four different groups on PN14. Each bar represents the mean ± *SD* for the groups. ^*^ indicates a significant difference from the control group, *p* < 0.05; # indicates a significant difference from the marginal ID group, *p* < 0.05.

**Figure 4:**
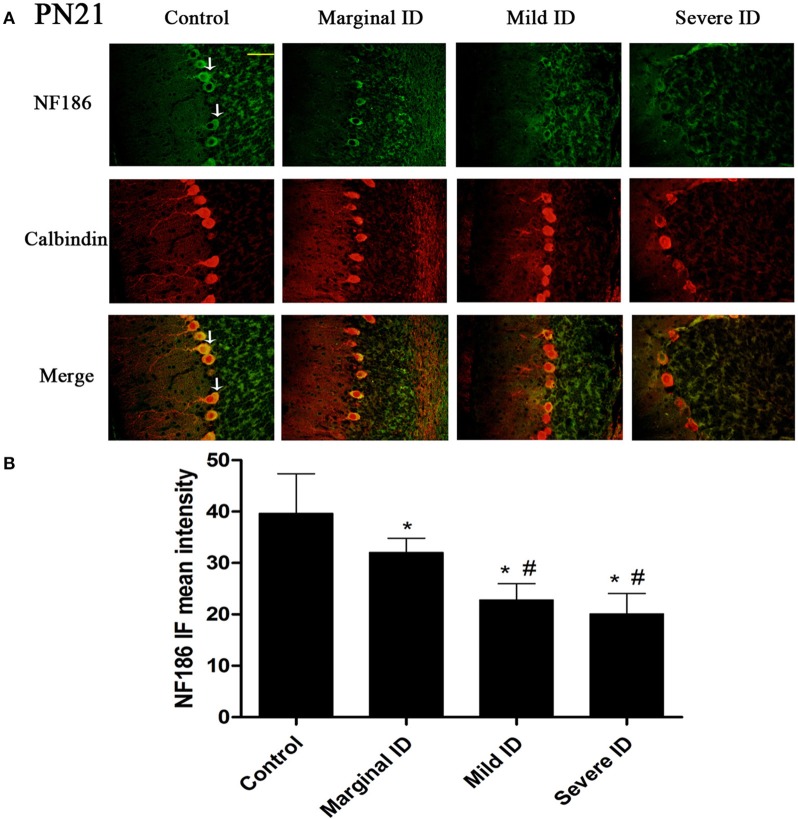
**Different degrees of ID decreased the levels of NF186 in cerebellar PCs on PN21**. Representative photomicrographs show fluorescent staining of anti- NF186(green) and anti-Calbindin-D-28 K (red) in the PCs on PN21 **(A)** (*n* = 5). The merged images show overlapping localization of these two proteins. NF186^+^ and Calbindin^+^ (arrow) show the pinceau. Scale bar = 100 μm. The bar graphs **(B)** show the results of the mean intensity of NF186 immunofluorescence in the four different groups on PN21. Each bar represents the mean ± *SD* for the groups. ^*^ indicates a significant difference from the control group, *p* < 0.05; # indicates a significant difference from the marginal ID group, *p* < 0.05.

As an isoform of the glutamic acid decarboxylase, GAD65 is found to coincide with cerebellar synaptogenesis and has been considered a specific marker of axon terminal on basket cells (Greif et al., 1991). NF186 is essential for pinceau organization (Buttermore et al., 2012). In addition, calbindin is a suitable marker of PCs. To study the effects of different degrees of ID on pinceau, the expression of GAD65 (Figures 1A, 2A) and NF186 (Figures 3A, 4A) in the cerebella PCs were analyzed. In the control group, the pinceau (arrow) labeled by GAD65^+^ Calbindin^+^ or NF186^+^ Calbindin^+^ was observed in cerebellar PCs. Compared with the control group, the mean intensity of GAD65 and NF186 positive staining in treated group were found to be significantly reduced on PN14 (Figures 1B, 3B; *p* < 0.05) and PN21 (Figures 2B, 4B; *p* < 0.05). These results illustrated that different degrees of ID could inhibit the development of pinceau in cerebellar PCs.

### Different degrees of ID reduced AnKG levels

**Figure 5:**
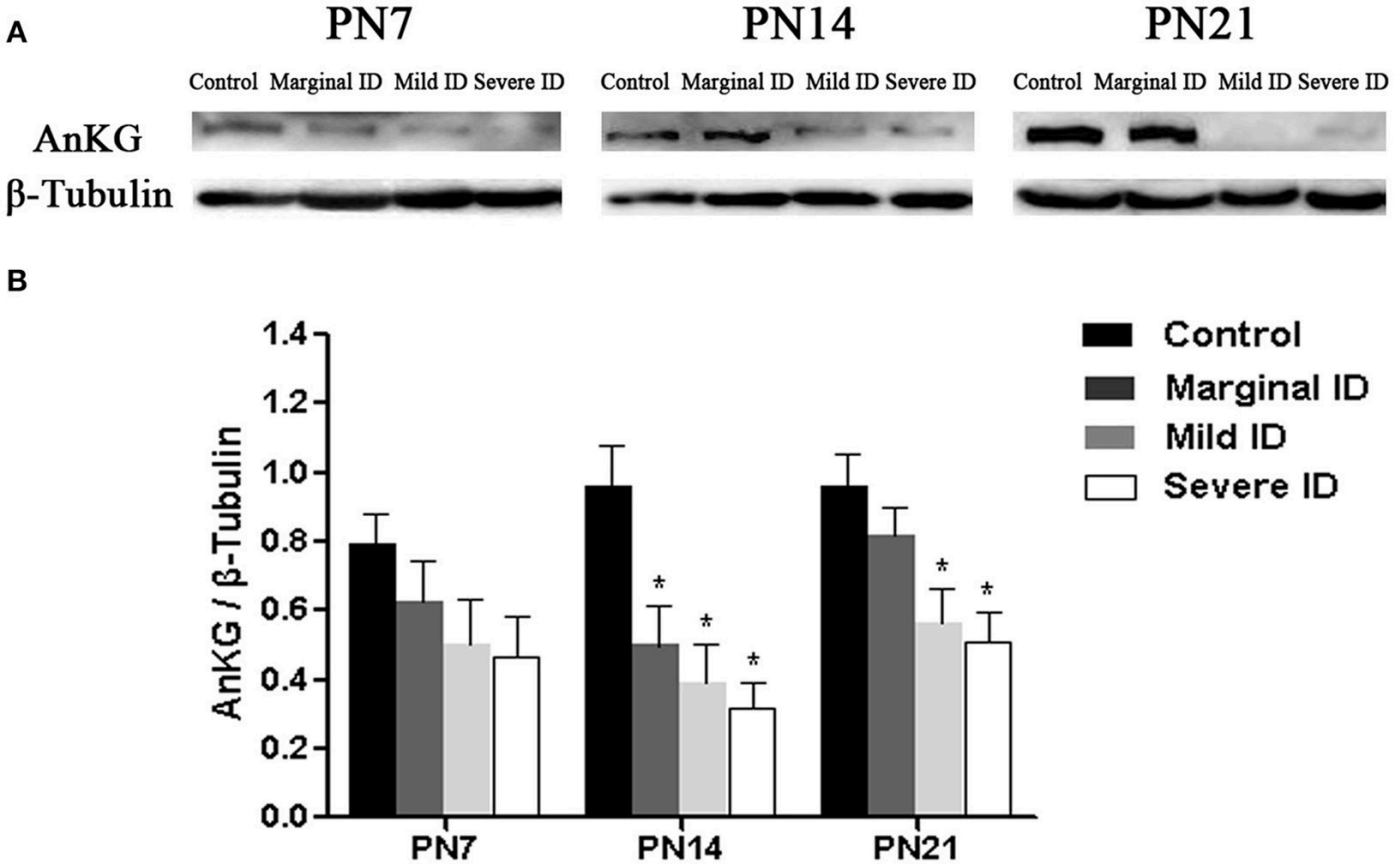
**Mild and severe ID reduced AnKG levels in cerebella**. Upper bands **(A)** depict representative findings as a result of developmental marginal, mild, and severe ID for rats. Lower bar graphs show the results of the semi-quantitative measurements of AnKG **(B)** on PN7, PN14, and PN21. Each bar represents the mean ± *SD* for the different groups. Within each time point, ^*^ indicates a significant difference from the control group, *p* < 0.05 (*n* = 5).

AnkG is critical for AIS organization, and is able to collect the other AIS components (Zhou et al., 1998; Hedstrom et al., 2008). So, we investigate the alteration of AnKG following different degrees of ID to study the development of cerebellar pinceau. Compared to the controls, the expressions of AnKG were significantly reduced in the mild and severe ID pups on PN14 and PN21. In addition, marginal ID significantly decreased the AnKG levels on PN14 (Figure 5; *p* < 0.05). While, significant difference was not observed in every group on PN7.

### Different degrees of ID reduced β4-spectrin levels

**Figure 6:**
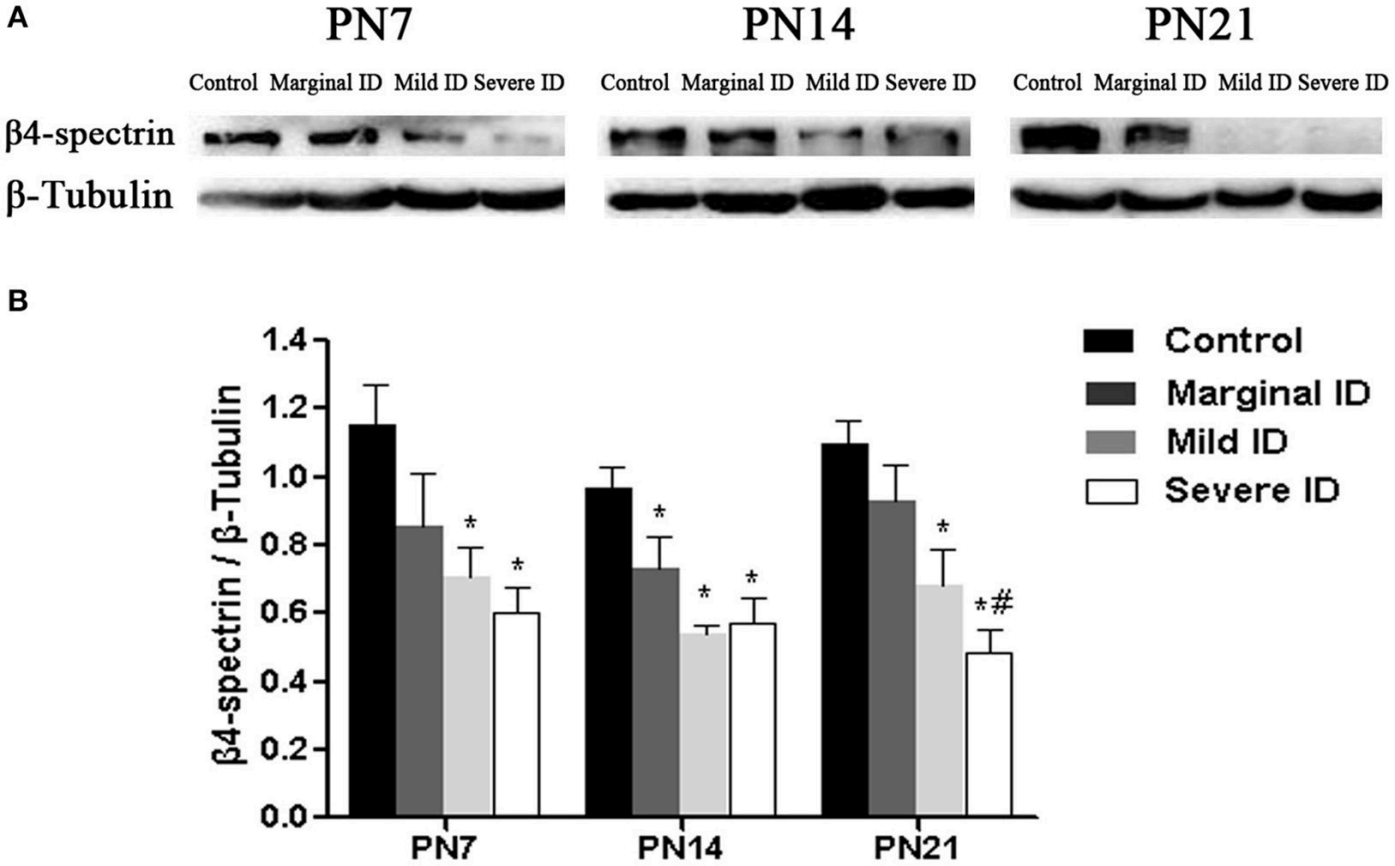
**Mild and severe ID reduced β4-spectrin levels in cerebella**. Upper bands **(A)** depict representative findings as a result of developmental marginal, mild, and severe ID for rats. Lower bar graphs show the results of the semi-quantitative measurements of β4-spectrin **(B)** on PN7, PN14, and PN21. Each bar represents the mean ± *SD* for the different groups. Within each time point, ^*^ indicates a significant difference from the control group, *p* < 0.05; # indicates a significant difference from the marginal ID group, *p* < 0.05 (*n* = 5).

β4-spectrin, as a specific scaffolding protein, is able to binds to AnkG to the actin cytoskeleton (Yang et al., 2007). Compared with the control group, significant decrease of β4-spectrin in cerebellar PCs was observed in mild and severe ID pups on PN7, PN14, and PN21 (Figure 6; *p* < 0.05). Moreover, marginal ID significantly decreased the β4-spectrin levels compared to the control group on PN14 (Figure 6; *p* < 0.05).

### Different degrees of ID reduced NF186 levels

**Figure 7:**
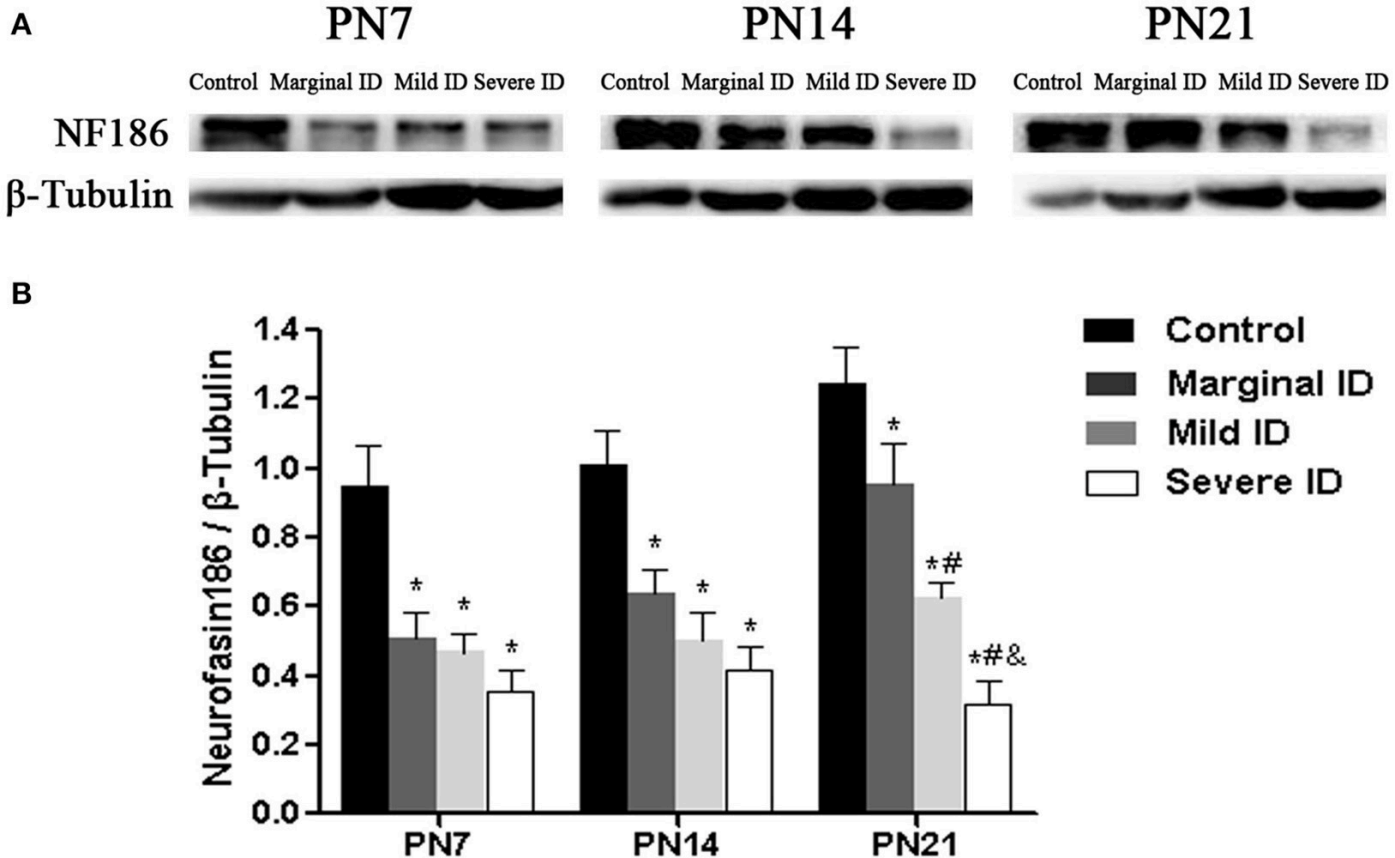
**Marginal, mild and severe ID reduced NF186 levels in cerebella**. Upper bands **(A)** depict representative findings as a result of developmental marginal, mild, and severe ID for rats. Lower bar graphs show the results of the semi-quantitative measurements of NF186 **(B)** on PN7, PN14, and PN21. Each bar represents the mean ± *SD* for the different groups. Within each time point, ^*^ indicates a significant difference from the control group, *p* < 0.05; # indicates a significant difference from the marginal ID group, *p* < 0.05; & indicates a significant difference from the mild ID group, *p* < 0.05 (*n* = 5).

It has been reported that NF186 is critical for localizing specific synaptic inputs to AIS and is necessary for its AIS targeting (Hedstrom et al., 2007). Therefore, we investigate the effects of marginal ID, mild ID, and severe ID on NF186 expression in PCs using immunoblot analysis. Compared to the controls, significant decrease of NF186 (Figure 7; *p* < 0.05) was found in the marginal ID, mild ID, and severe ID pups on PN7, PN14, and PN21. In addition, the significant decrease of NF186 levels were observed in the mild ID and severe ID groups compared with the marginal ID on PN21 (Figure 7; *p* < 0.05).

### Different degrees of ID reduced NrCAM levels

**Figure 8:**
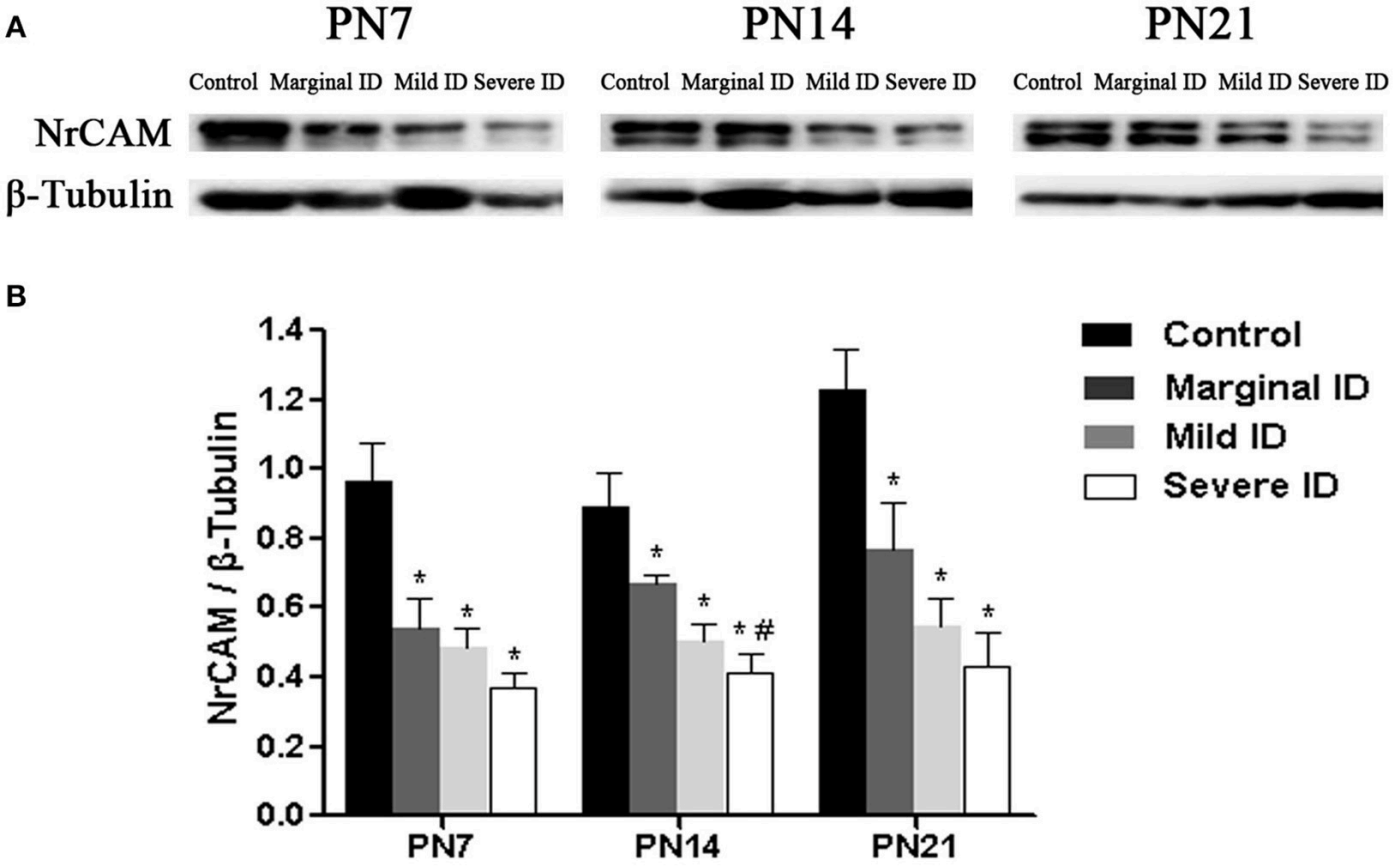
**Marginal, mild and severe ID reduced NrCAM levels in cerebella**. Upper bands **(A)** depict representative findings as a result of developmental marginal, mild, and severe ID for rats. Lower bar graphs show the results of the semi-quantitative measurements of NrCAM **(B)** on PN7, PN14, and PN21. Each bar represents the mean ± *SD* for the different groups. Within each time point, ^*^ indicates a significant difference from the control group, *p* < 0.05; # indicates a significant difference from the marginal ID group, *p* < 0.05 (*n* = 5).

Extracellular signaling molecule NrCAM is another L1 family members enriched in the AIS (Ogawa and Rasband, 2008). Relative to the controls, a significant decrease of NrCAM was observed in the marginal, mild and severe ID group at all time points (Figure 8; *p* < 0.05). Only on PN14, there was a significant difference between the marginal ID group and the severe ID group.

## Discussion

Iodine deficiency is a worldwide problem of immense magnitude affecting 2 billion of the world's population, which leads to dysfunction of central nervous system and impairments of motor and psychomotor function (Zimmermann and Andersson, 2012). Although universal salt iodization has taken several decades, there are also a large percentage of women of reproductive age suffering from marginal or mild ID (Ferreir et al., 2014; Méndez-Villa et al., 2014). Therefore, we should concern about the impacts on the fetus following maternal mild and marginal ID. In the present experiment, maternal different degrees of ID rat models had been successfully established by treating with an iodine deficient diet during pregnancy and lactation. And the postnatal development of pinceau in cerebellar PCs and the underlying mechanisms involved had become the focus of our attention.

Cerebellar formation is principally in the postnatal period of rodents (Velázquez-Zamora et al., 2011), which required sufficient TH supply (Melse-Boonstra and Jaiswal, 2010). It has been widely believed that adequate TH during the critical neurodevelopment period regulates cerebellar development and functional formation, including in dendrite growth and branching, axon elongation, synaptogenesis, and neuronal proliferation, differentiation and migration, via genomic and non-genomic effects (König and Moura Neto, 2002; Ausó et al., 2004; Cheng et al., 2010; Patel et al., 2011; Chen et al., 2012). Especially, it has been confirmed that T_4_ is critical to cerebellar functional organization and development (Farwell et al., 2005; Manzano et al., 2007; Shimokawa et al., 2014; Oyanagi et al., 2015). Through non-genomic pathway, T_4_ promotes cytoskeletal actin polymerization and integrin interaction with laminin in neural cells, and alters activity of iodothyronine deiodinase, which leads to the activation of mitogen-activated protein kinase (MAPK) signal and phosphorylation of thyroid hormone receptor (Di Liegro, 2008; Cheng et al., 2010). So, it is speculated that deficiency of T_4_ induced by different degrees of ID may contribute to alteration of protein involved in neurodevelopment, especially some cytoskeletal proteins, which ultimately resulted in impairment of cerebellar development and formation. Our previous research has confirmed that ID could reduce dendritic growth and branching of PCs, decrease the proliferation of CGNPs, inhibit the differentiation of granular cells, and reduce the parallel fiber-purkinje cell synapses (Wang et al., 2014a,b, 2016; Dong et al., 2016). It has been identified that these abnormal cerebellar developments induced by deficient TH results in impaired motor memory and behavioral changes, such as anxiety, abnormal sports coordination, decreased autonomic activities, and so on (Berbel et al., 2009; Manto and Jissendi, 2012; Galliano et al., 2013; Koibuchi, 2013). And electrophysiological experiments have also confirmed the decrease of neurotransmitter release and impairments of synapses following deficient TH (Koibuchi, 2013).

PCs are the main cells of the cerebellar cortex, and are the only efferent neuron from the cerebellum to the other encephalic regions (Gutierrez et al., 2011). Purkinje neurons receive the inhibitory GABAergic inputs from basket cells and astrocytes (Bayer and Altman, 1987). The astrocytes selectively innervate PCs dendrites and spines. While the basket cells show highly specific and are anchored AIS of PCs to form a specialized structure, which is called pinceau synapses. Inhibition of the Purkinje AIS is achieved through GABAergic synapses releasing the inhibitory neurotransmitter GABA to dampen Purkinje cell excitability (Purves et al., 2004; Huang et al., 2007). Importantly, the pinceau is crucial to PCs development and function. It has been demonstrated that impairment of pinceau functional organization resulted in ataxia, which is a consequence of PCs dysfunction (Xie et al., 2010). After the 2 weeks following birth, the axon terminal of basket cells surrounded PCs and extended to AIS, which is accompanied with the distribution of GABAergic neuron on PCs (Ango et al., 2004). So, in this study, we have shown that developmental mild and severe ID significantly inhibited the development of pinceau in cerebellar PCs on PN14, and PN21. It is worth noting that, marginal ID also inhibited the development of pinceau in cerebellar PCs.

The AIS is a unique neuronal subregion involved in the initiation of action potentials and the command of axonal identity, which plays a crucial role in the developmental formation and long-term maintenance of the pinceau (Grubb and Burrone, 2010; Buttermore et al., 2012). AnkG has been identified to be targeted to the Purkinje AIS, which is considered to be an adaptor protein that can link membrane proteins to the spectrin/actin cytoskeleton (Zhou et al., 1998; Bennett and Baines, 2001; Boiko et al., 2007). An *in vivo* research has shown that transgenic mice lacking AnkG also do not develop an AIS (Zhou et al., 1998). And cultured neurons knocked down AnkG never form a normal AIS (Hedstrom et al., 2008). Furthermore, knockdown of AnkG using shRNA led to deficiency of all other AIS components in the AIS, including NF186, NrCAM, β4-spectrin and so on (Hedstrom et al., 2007). More importantly, lack of AnkG also resulted in disrupted AIS formation and axonal polarity, which leads to abnormal organization of the pinceau (Sobotzik et al., 2009). In this study, our data showed that AnkG expressions significantly reduced in the offspring with mild and severe ID. And the slightly decreased AnkG expressions were observed in the marginal ID pups. Therefore, it is speculated that different degrees of ID inhibit pinceau development in cerebellum by down-regulating AnkG.

β4-spectrin is an actin-binding cytoskeletal protein, which is a member of the spectrin family and binds to AnkG and the actin cytoskeleton (Yang et al., 2007; Grubb and Burrone, 2010). *In vivo* studies showed that neurons lacked the clear AIS in β4-spectrin KO mice (Zhou et al., 1998; Lacas-Gervais et al., 2004). So, it is speculated that β4-spectrin may also be critical to the development of AIS. Importantly, severe auditory and motor disorders were found in the animals carried a loss-of-function mutation in β4-spectrin, which further confirmed the importance of β4-spectrin in AIS development (Parkinson et al., 2001). Our results showed that β4-spectrin levels significantly decreased in mild and severe ID pups. However, as to marginal ID, β4-spectrin levels also slightly reduced in the offspring. So, the down-regulation of β4-spectrin levels may be the possible mechanism of inhibiting pinceau formation in cerebellum.

In addition, a variety of CAMs including two members of the L1 family of CAMs, NF186 and NrCAM, are abundant in the AIS (Ogawa and Rasband, 2008). NF186 on PCs serves as a substrate for basket axon growth, and a subcellular NF186 gradient may further provide a directional cue from soma to AIS. On purkinje soma, neurofascin is displayed in dimeric or monomeric forms with low-affinity cell adhesion. And at AIS and in association with AnkG and β4-spectrin, highly crosslinked oligomeric forms of neurofascin ensure the high-affinity of cell adhesions, which could promote pinceau synapse formation (Ango et al., 2004). Furthermore, NF186 is essential for the function organization and long-term maintenance of the AIS (Hedstrom et al., 2008; Zonta et al., 2011), and linking AIS cytoskeleton to the extracellular matrix (Hedstrom et al., 2007). Our results showed that marginal, mild and severe ID significantly reduced NF186 and NrCAM expression on PN7, PN14, and PN21. These findings suggested that different degrees of ID could result in down-regulated NF186 and NrCAM expression, which could reduce AIS stability of Purkinje cells and inhibit pinceau formation in cerebellum.

In this study, we observed a reduction of pinceau synapses in cerebellar caused by marginal, mild and severe ID. We also confirmed that the alterations of AnkG, NF186, NrCAM, and β4-spectrin, which closely related to AIS stability and pinceau development, led to the decrease of pinceau synapses in cerebellar PCs following maternal marginal, mild and severe ID in the rat offspring. We therefore speculated that maternal different degrees of ID might result in the impairments of cerebellar neurodevelopment and function organization, attributing to the reduction and inhibition of pinceau formation in rat offspring, which might be due to the down-regulation of AnkG, NF186, NrCAM, and β4-spectrin.

Our findings further emphasized that even slight deficiency of iodine may be harmful to the neurodevelopment of offspring, which ultimately resulted in psychomotor disorders. While, it is worth noting that there are a large number of women of child bearing age in the situation of marginal ID, with the UIC of 100–150 μg/l (Ferreir et al., 2014; Méndez-Villa et al., 2014). Furthermore, even in developed countries, the pregnant women are still in the situation of mild to moderate ID (Bath and Rayman, 2013; Clifton et al., 2013). Therefore, it is necessary to screen the thyroid function and adequate iodine supplementation during pregnancy and lactation, which may reduce the risk of neurodevelopmental delay and cognitive and psychomotor dysfunction in the fetus or infants.

## Author contributions

JD and JC conceived and designed the study. HS and ML carried out the whole experiment. JD and HS analyzed the results and wrote the manuscript. All authors participated in its revision, and approved the final manuscript.

### Conflict of interest statement

The authors declare that the research was conducted in the absence of any commercial or financial relationships that could be construed as a potential conflict of interest.
